# Spatial and Environmental Drivers of Summer Growth Variability and Adaptive Mechanisms of *Euphausia crystallorophias* in the Amundsen Sea and Its Adjacent Regions

**DOI:** 10.3390/ani15223345

**Published:** 2025-11-20

**Authors:** Jialiang Yang, Lingzhi Li, Shuai Li, Guoqing Zhao, Xin Rao, Shuai Chen, Hewei Liu, Fengyuan Shen, Hongliang Huang, Ziyi Wang

**Affiliations:** 1 East China Sea Fisheries Research Institute, Chinese Academy of Fishery Sciences, Shanghai 200090, China; yangjl@ecsf.ac.cn (J.Y.); lishuai@ecsf.ac.cn (S.L.); zgq617717@163.com (G.Z.); raoxin128@gmail.com (X.R.); yangpu79@hotmail.com (S.C.); hwliu77@126.com (H.L.); fyshen0817@126.com (F.S.); ecshhl@163.com (H.H.); wzy18018994337@163.com (Z.W.); 2 Key Laboratory of Oceanic and Polar Fisheries, Ministry of Agriculture and Rural Affairs, Shanghai 200090, China

**Keywords:** ice krill, length–weight relationship, growth strategy, generalized additive model, redundancy analysis, Amundsen sea and adjacent regions

## Abstract

Ice krill (*Euphausia crystallorophias*) play a key role in the Antarctic coastal ecosystem, yet its spatial growth variability remains poorly understood. This study examined 5298 krill individuals from 52 stations across the Amundsen Sea, transitional waters, and the Ross Sea, collected between 2020 and 2024. Length–weight relationships (LWR) were constructed to derive the condition factor *a* and the allometric growth exponent *b*, followed by regional comparisons and environmental response analyses using boxplots, redundancy analysis (RDA), and generalized additive models (GAM). Boxplots revealed that *a* was significantly higher in the Amundsen Sea and transitional zone than in the Ross Sea, while *b* was highest and most variable in the Amundsen Sea. RDA indicated that *a* was primarily associated with depth, latitude, mean temperature, and mean salinity, whereas *b* was influenced by sea surface temperature, chlorophyll-*a*, sea ice concentration, and longitude. GAM further showed nonlinear responses of *a* to mean temperature, mean salinity, and depth, with peaks near −0.5 °C, 34.2 PSU, and 3500 m, respectively. These results suggest that krill in deep, cold, and less-productive transitional zone allocate more energy to body condition (high value *a*), while those in warmer, moderately productive regions like the Amundsen Sea invest more in structural growth (high value *b*). This study provides new insights into the environmentally driven growth strategies of ice krill and contributes to understanding its ecological adaptability under changing climatic and oceanographic conditions.

## Simple Summary

Ice krill are a key component of the Antarctic food web, but scientists still lack a full understanding of how different environmental conditions affect their growth. This study focused on the Amundsen Sea and adjacent Antarctic waters to examine how water temperature, salinity (saltiness), sea ice cover, and location influence ice krill growth in summer. The researchers found that environmental differences led to noticeable changes in krill growth. For example, in slightly warmer waters with abundant food (often near melting sea ice), ice krill grew faster and increased in length. In colder conditions with less food available, they gained weight (stored energy) instead of growing longer. This demonstrates that ice krill can adjust their growth strategy in response to different environmental conditions. Understanding this flexibility is important because it helps scientists predict how krill and the Antarctic ecosystem will respond to a warming climate and changing sea ice conditions. Ice krill are a vital food source for penguins, seals, and whales, and they also help store carbon in the ocean. Therefore, understanding their growth helps us anticipate broader effects of climate change in Polar Regions.

## 1. Introduction

The Antarctic ice krill (*Euphausia crystallorophias*), a key species in the Southern Ocean marine ecosystem, predominantly inhabits coastal waters, with particularly dense populations observed in the Amundsen Sea. Although phylogenetically close to the Antarctic krill (*Euphausia superba*), this cryopelagic specie plays a crucial trophic role in the food web by transferring energy from primary producers to apex predators, including notothenioid fish, penguins, and cetaceans [1]. Growth parameters of ice krill provide valuable insights into both individual fitness and broader ecosystem responses to environmental perturbations [2]. Over recent decades, the Southern Ocean has undergone substantial environmental changes, including a 0.17 °C per decade increase in sea surface temperature, a 1.5% annual reduction in sea ice extent, and poleward shifts in primary productivity patterns [3]. These changes are likely to influence the growth, survival, and biogeographic distribution of ice krill populations. Previous studies have revealed significant correlations between euphausiid growth and environmental factors such as temperature, salinity, and chlorophyll-a concentration [4,5]. However, the specific growth responses and adaptive strategies of ice krill to environmental gradients remain poorly understood. The length–weight relationship (LWR) is a fundamental tool in fisheries and ecological studies for evaluating organismal growth and nutritional condition. It is typically described by the power function: *W* = *a*·*L^b^*, where *W* is body weight, *L* is body length, *a* is the condition factor, and *b* is the allometric growth exponent. While this model is widely applied in finfish biology [6], it is also extensively used in crustacean studies to assess individual health, population status, and environmental adaptation potential [7].

The Amundsen Sea and its adjacent regions, located in the Pacific sector of the Southern Ocean, is a climatically sensitive marginal sea characterized by pronounced hydrographic variability. In recent years, the region has experienced significant sea ice retreat (12% per decade) and a 0.4 °C increase in temperature, along with changes in mixed layer depth and primary productivity patterns [8]. These environmental perturbations likely influence the growth and spatial distribution of ice krill. However, studies focusing specifically on the length–weight dynamics of ice krill in the Amundsen Sea remain limited. Understanding how environmental gradients shape their growth condition is essential for predicting population trends and ecological resilience. Although substantial progress has been made in the biological and ecological understanding of Antarctic krill, research on ice krill particularly under fine-scale environmental variability remains scarce. As an ice-associated, nearshore species with strong cold adaptation, ice krill are highly sensitive to changes in sea ice cover, water mass structure, and food availability. Their life history strategies may differ significantly from other euphausiids [9]. A comprehensive synthesis of their life cycle characteristics, trophic behavior, and distributional ecology was provided by Siegel [10]. Furthermore, euphausiids play an essential role in biogeochemical cycling, contributing to carbon flux through egestion and post-mortem sinking [11]. The physical structure of the Amundsen Sea, especially its complex coastal circulation and water mass transformation processes [12], may also mediate food availability and habitat suitability for krill.

Recent studies have attempted to model krill development in relation to environmental variables. For example, Meyer [13] demonstrated that rising temperatures significantly alter larval development trajectories, affecting recruitment and life cycle duration. Similarly, Bahlburg [14] highlighted the importance of model selection when predicting krill growth under changing environmental conditions. Environmental drivers, such as temperature and food availability, play key roles in shaping growth outcomes and determining the shifting ecological niches of Antarctic krill. However, current research on the growth status of ice krill has primarily focused on individual traits [15], population distribution [16], or reproductive maturity. Few studies have quantitatively linked individual-level biometric data (length and weight) of ice krill with environmental gradients to explore fine-scale spatial variation in krill growth. The spatial variation in LWR parameters, particularly the condition factor *a* and allometric exponent *b*, can serve as sensitive indicators of physiological adaptation and environmental stress. These parameters help elucidate energy allocation strategies and trade-offs between growth and reproduction [6,17]. Therefore, quantifying how LWR metrics vary across ecological gradients offers an effective approach to infer habitat suitability and physiological plasticity in response to environmental forcing.

This study aims to address these knowledge gaps by investigating the spatial variation in the growth patterns of ice krill across environmental gradients in the Amundsen Sea and adjacent regions, including the Ross Sea and the transitional zone between the two seas. Based on field samples collected during the 36th to 40th Chinese Antarctic scientific expeditions support by Ministry of Natural Resources of the People’s Republic of China, we have combined individual-level length and weight measurements with environmental and spatial data to develop length–weight relationship (LWR) models and analyze their spatial patterns. Through this work, we aim to improve our understanding of the ecological strategies and environmental responses of ice krill under rapid climatic change and to provide scientific insights for ecosystem-based management and conservation efforts in the Southern Ocean.

## 2. Materials and Methods

### 2.1. Study Area

**Figure 1:**
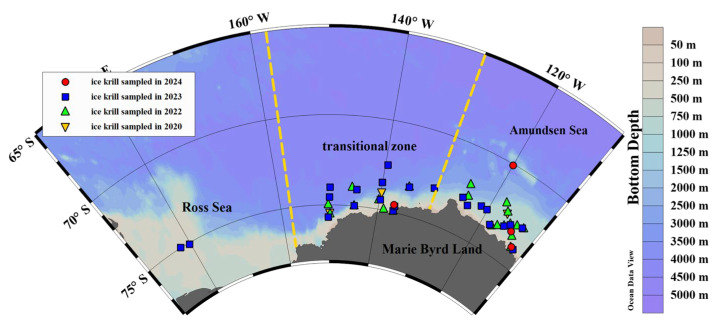
Survey area showing the station of ice krill sampled in 2020~2024 in Amundsen Sea, transitional zone between Amundsen-Ross Sea, and Ross Sea.

This study was conducted in the Amundsen Sea (Figure 1), a marginal sea off West Antarctica, which is characterized by complex oceanographic dynamics and pronounced environmental gradients. In recent decades, the region has undergone rapid climate-driven changes, including significant sea ice retreat and warming. These changes make it a critical area for investigating the ecological responses of polar marine organisms [8]. In addition, previous studies have identified a mechanism by which ice krill populations are transported from the Amundsen Sea to adjacent waters, driven by the Antarctic Coastal Current. To fully investigate the drivers of growth variation in this species, our study area was expanded to include the Amundsen Sea, the transitional zone between the Amundsen and Ross Seas, and the Ross Sea itself [15].

### 2.2. Sample Collection and Preservation

Ice krill samples were collected during the austral summers (January to March) from 2020 to 2024, as part of China’s 36th to 40th Antarctic scientific expeditions. The surveys were conducted aboard the Chinese polar research vessels Xuelong (in 2020, 2022, and 2023) and Xuelong 2 (in 2024), covering data from four expedition cruises. A total of 52 sampling stations were established, spanning ecological zones from nearshore to offshore areas within the Amundsen Sea and its adjacent regions (Figure 1). At each station, a rectangular midwater trawl (RMT8) with a mouth area of 8 m^2^ and a mesh size of 500 µm was deployed, sampling from the surface to a depth of 250 m to cover the primary vertical distribution range of ice krill. Each tow lasted approximately 40 min, and both cable deployment and retrieval speeds were kept consistent across stations to ensure sampling comparability. Although no filtered volume was directly measured and no replicate tows were conducted, the uniform towing procedure provided consistent and comparable biometric data. Upon retrieval, bulk samples were pre-sorted onboard and immediately preserved at −20 °C for subsequent laboratory analysis.

### 2.3. Biological Measurements

In the laboratory, specimens were morphologically identified as ice krill according to the classification criteria of Siegel [10]. For each specimen, the following biometric parameters were measured as follows: Total Length (*TL*): Measured from the anterior margin of the eye to the posterior end of the telson using digital calipers accurate to 0.1 mm; Wet Weight (*W*): Determined using an analytical balance with 0.1 mg precision after gently blotting excess moisture from the body surface. At least 100 individuals were measured per station to ensure robust statistical analysis. The length–weight relationship (LWR) was modeled using the allometric power function as follows:

(1) $$W=a\cdot TL^{b}$$

where *a* is the condition factor and *b* is the allometric growth exponent [6].

### 2.4. Environmental Data Acquisition and Processing

Environmental parameters were collected concurrently with biological sampling. These included the following variables: sea surface temperature (SST), sea surface salinity (SSS), mean temperature (Temp) and mean salinity (Salinity) above 200 m, Chlorophyll-*a* (Chl-*a*). All parameters were measured in situ using a Sea-Bird CTD profiler (SBE 911 plus). The data were extract by SBE Data Processing software (version 7.26.7) and were further processed and analyzed for hydrographic characteristics using Ocean Data View software (version 4.7.10). Sea ice concentration (SIC) data with a spatial resolution of 25 km, were obtained from satellite remote sensing products provided by the National Snow and Ice Data Center (NSIDC). SIC values corresponding to each sampling station were extracted using spatial and temporal interpolation in Python, based on the geographic coordinates and sampling dates; the topographic data including bottom depth, were recorded using the EA600 echo sounder system onboard the survey vessel. These environmental variables have been shown to significantly influence ice krill distribution and growth in polar ecosystems [18].

### 2.5. Statistical Analyses

#### 2.5.1. Length–Weight Relationship Modeling

To linearize the LWR model, both length and weight data were log-transformed:

(2) Log(W)=Log(a)+b⋅Log(TL)

For each station, linear regression models were fitted to estimate the parameters *a* and *b*. Separate models were established for all samples combined, as well as for ice krill populations within each region. The coefficient of determination (R^2^) was used to evaluate the model fit.

#### 2.5.2. Spatial Variation Analysis

Boxplot analysis was used to explore regional variation in the LWR parameters of ice krill, with sampling region defined as the grouping variable. Outliers at each station were identified and removed using the standard interquartile range (IQR) method. The medians and interquartile ranges of the LWR parameters (*a* and *b*) were then compared across regions to evaluate spatial differences in ice krill growth patterns.

#### 2.5.3. Environmental Effects on Growth

To examine the effects of environmental variables on krill growth, multiple linear regression models were developed using LWR parameters *a* and *b* as response variables. Predictors included SST, salinity, Chl-*a*, and SIC. Prior to modeling, multicollinearity among predictor variables was assessed using the variance inflation factor (VIF), and variables with VIF value greater than 5 were excluded from the final model. Additionally, redundancy analysis (RDA) was employed to explore the relationships among krill growth metrics and environmental gradients. All analyses were conducted in R-Project, with RDA implemented using the “vegan” package [19]. The environmental variable most closely aligned with the primary RDA axis was subsequently selected to build a generalized additive model (GAM). The selection of environmental predictors was informed by previous studies on krill biology and broader ecological evidence demonstrating that temperature and food availability jointly influence growth responses in polar ectotherms [20]. Notably, variations in individual euphausiids weight may also reflect differences in lipid storage and recent feeding success [21], which are influenced by environmental heterogeneity such as SST, salinity, Chl-*a* [22]. Furthermore, marginal ice zones, such as those in the Amundsen Sea, often function as biological hotspots for phytoplankton and zooplankton production, thereby providing favorable foraging conditions for euphausiids [23,24].

## 3. Results

### 3.1. Environmental Characteristics in Survey Area

**Figure 2:**
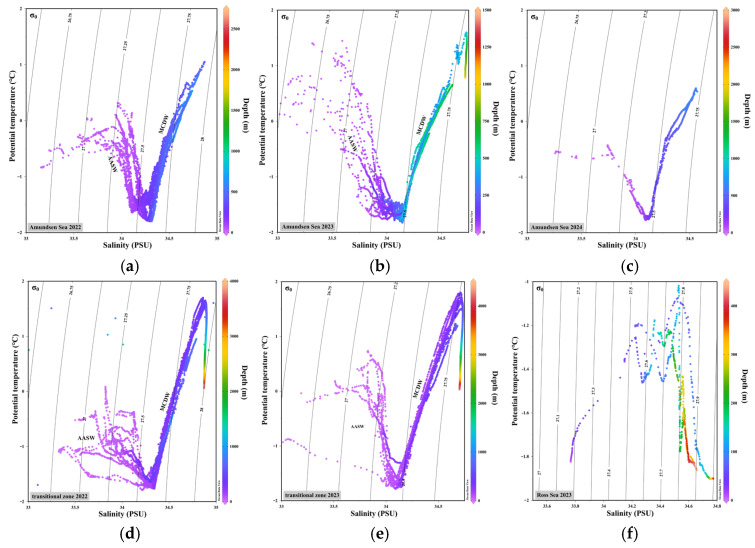
θ-S diagrams of water mass structure in the Amundsen Sea (AMS), Amundsen-Ross Sea transition zone (RAS), and Ross Sea (RS) during the austral summers of 2022~2024: (**a**) AMS in 2022; (**b**) AMS in 2023; (**c**) AMS in 2024; (**d**) RAS in 2022; (**e**) RAS in 2023; (**f**) RS in 2023.

The θ-S diagrams from the Amundsen Sea (AMS), the Amundsen-Ross Sea transition zone (RAS), and the Ross Sea (RS) reveal distinct spatial and temporal patterns in water mass structure between 2022 and 2024 (Figure 2). Across all regions, a characteristic three-layer stratification was observed, consisting of cold, fresh Antarctic Surface Water (AASW) in the upper layer, a transitional layer in the middle, and warm, saline Modified Circumpolar Deep Water (MCDW) in the deep layer. In the AMS, a distinct V-shaped θ–S curve was consistently observed over all three years. The surface water mass (θ < 0 °C, S < 34 PSU) was consistently present, while the deep water was dominated by MCDW. Between 2022 and 2024, the MCDW signal gradually intensified, with θ increasing to 1.4–1.5 °C and salinity approaching or exceeding 34.8 PSU. These changes suggest a stronger and deeper intrusion of warm, saline water masses; In the RAS, a strong MCDW signature was already observed in 2022 (θ > 1.0 °C, S > 34.7 PSU), and became more prominent in 2023. The upward extension of the MCDW branch in the θ-S diagram indicates an elevated influence of deep water. In contrast, the surface layer appeared more scattered and unstable, likely influenced by wind-driven mixing or input from sea ice melt; Unlike the AMS and RAS, the θ-S profile for the RS in 2023 exhibits atypical thermal stratification, with temperature increasing with depth in the upper layer before decreasing in deeper waters. This inverted structure likely reflects reduced presence of MCDW and the combined effect of surface heat accumulation and remnants of subsurface winter water over the shallow continental shelf. Overall, the strength of MCDW intrusion decreased from RAS to AMS to RS, whereas water column stratification followed the opposite trend (RS > AMS > RAS).

**Figure 3:**
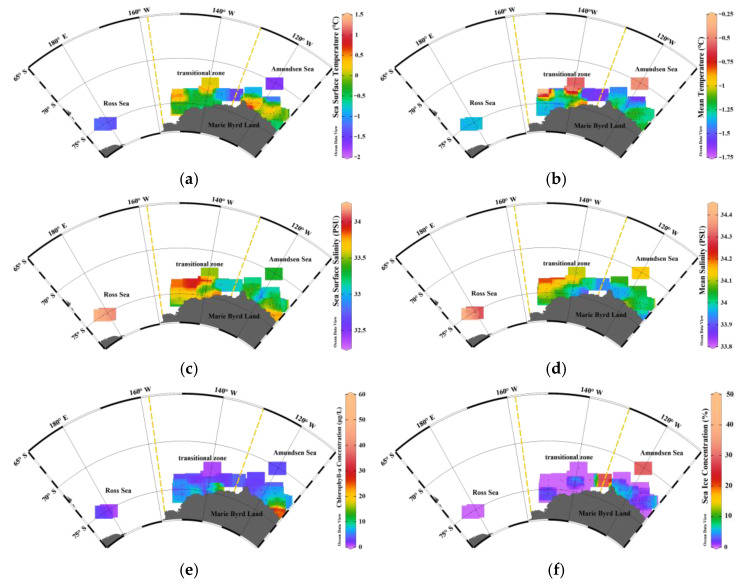
Spatial patterns of six environmental variables: (**a**) sea surface temperature (SST); (**b**) mean temperature of the upper 200 m (Temp); (**c**) sea surface salinity (SSS); (**d**) mean salinity of the upper 200 m (Salinity); (**e**) chlorophyll-*a* concentration (Chl-*a*) and (**f**) sea ice concentration (SIC) across the Amundsen Sea (AMS), transitional zone (RAS), and Ross Sea (RS).

The spatial distributions of key environmental variables showed clear horizontal gradients across the AMS, RAS, and RS (Figure 3). SST exhibited a distinct east-to-west gradient, with the warmest surface waters located in the eastern AMS (0 to 1.5 °C), intermediate values in the RAS (−0.5 to 0.5 °C), and the coldest SST values in the RS (generally below −1.0 °C). In contrast, SSS was highest in the RS (>34.0 PSU), intermediate in the RAS, and lowest in the AMS (~33.5 PSU). SIC increased from west to east, with low concentrations in the RS (<15%), moderate levels in the RAS (10–30%), and the highest values in the eastern AMS (>40%). Mean temperature displayed a trend similar to that of SST, with colder conditions in the RS (<−1.5 °C) and progressively warmer waters toward the AMS. Chl-*a* was spatially heterogeneous, with peak values observed in the coastal AMS region (>20 μg/L), while the RS and RAS remained relatively low (<10 μg/L). Mean salinity mirrored to the distribution of SSS, with the RS showing the highest values (34.4 PSU), decreasing gradually through the RAS to the AMS (as low as 33.9 PSU). These spatial patterns reflect strong environmental gradients along both longitudinal and coastal-offshore axes, providing a critical foundation for interpreting regional differences in ice krill growth and physiological condition.

### 3.2. Length–Weight Relationship of Ice Krill

**Figure 4:**
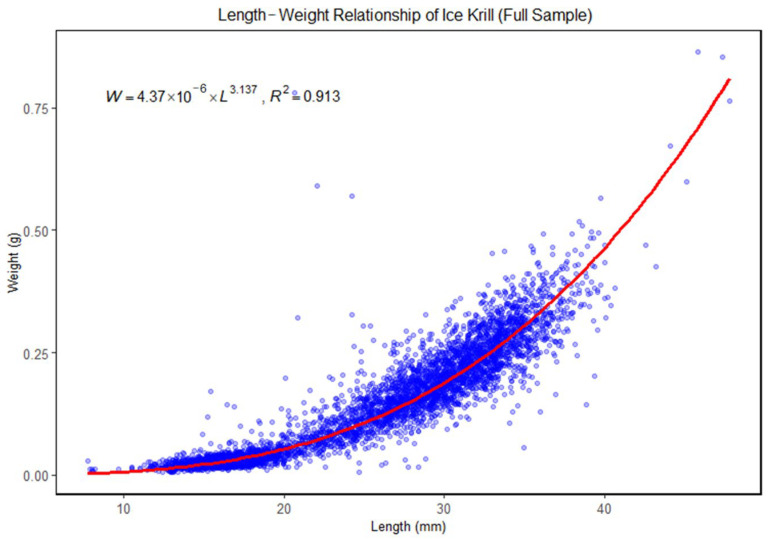
Length–weight relationship (LWR) for ice krill based on the full sample from the study region. The coefficient of determination of *R*^2^ = 0.913, indicating a strong positive correlation. The red curve represents the non-linear least squares fit to the data.

The length–weight relationship (LWR) for ice krill was derived based on all samples collected across the study area. The fitted model is expressed as follows:

(3) $$W=4.37\times 10^{-6}\times L^{3.137}$$

where *W* represents the wet weight (*g*) and *L* is the total length (mm). The coefficient of determination was high (*R*^2^ = 0.913), indicating a strong correlation between total length and weight across the full sample. The estimated allometric exponent *b* = 3.137 indicates positive allometric growth, suggesting that individuals gain weight at a greater rate than length during development (Figure 4).

**Figure 5:**
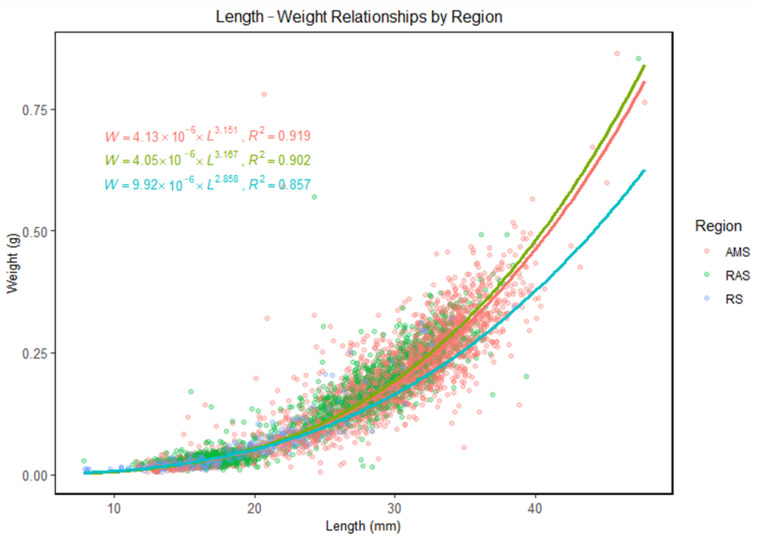
Length–weight relationship (LWR) of ice krill in different region.

The length–weight relationships of ice krill were further examined by region. In the Amundsen Sea (AMS), the fitted LWR model was $W=4.13\times 10^{-6}\times L^{3.151}$ with a coefficient of determination of *R*^2^ = 0.919. In the transition region between the Amundsen Sea and Ross Seas (RAS), the fitted LWR model was $W=4.05\times 10^{-6}\times L^{3.167}$, with *R*^2^ = 0.902. In the Ross Sea (RS), the fitted model was $W=9.92\times 10^{-6}\times L^{2.858}$, and the coefficient of determination was *R*^2^ = 0.857. The data points and regression curves for each region are illustrated in Figure 5.

### 3.3. Regional Distributions of LWR Parameters Across Sampling Areas

**Figure 6:**
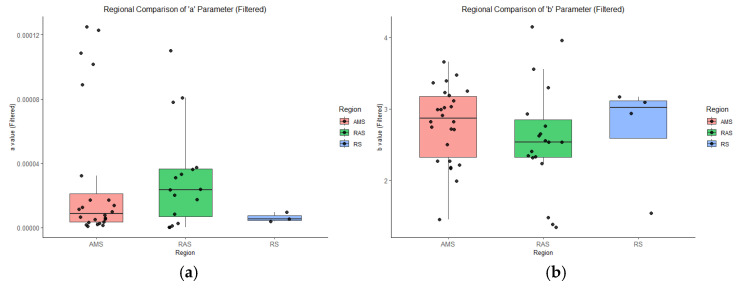
Boxplots of the LWR parameters of ice krill by region (AMS: Amundsen Sea; RAS: Amundsen-Ross Sea transitional zone; RS: Ross Sea): (**a**) parameters *a* (condition factor); (**b**) parameters *b* (allometric exponent). Extreme outliers were removed using the interquartile range method. Boxes represent interquartile ranges, the horizontal line indicates the median, and black dots represent individual station-level estimates.

After removing extreme outliers using the IQR method, the distributions of the LWR parameters *a* and *b* showed clear regional differences (Figure 6). The condition factor *a* exhibited relatively low values in the Ross Sea, with a narrow interquartile range and minimal variability. In contrast, the Amundsen Sea and the transitional zone between the Amundsen and Ross seas displayed higher median values and greater dispersion of *a*.

For the allometric growth exponent *b*, the AMS region exhibited the highest variability, with a median value exceeding 2.8. The RAS group showed a slightly lower median and broader dispersion. In contrast, the RS group displayed a concentrated distribution centered around a higher median value (≈3.0), but with less variability than AMS or RAS.

### 3.4. RDA of LWR Parameters with Environmental and Spatial Variables

Prior to conducting the RDA, a VIF analysis was performed on all candidate environmental and spatial variables to avoid potential bias due to multicollinearity (Table 1). The results showed that all variables had VIF values below the standard threshold of 5 when evaluated in regression models for the LWR parameters *a* and *b*, indicating low collinearity among predictors. The highest VIF was observed for mean salinity at 0–200 m, which remained within acceptable limits, reaching 3.637 and 3.531 for *a* and *b*, respectively. Therefore, the following nine variables were retained as explanatory variables in the subsequent RDA: SST, SSS, Chl-*a*, SIC, mean temperature (Temp) and salinity (Salinity) at 0–200 m, bottom depth, latitude, and longitude. These variables were used to assess their effects on the spatial variation in ice krill LWR parameters.

**Figure 7:**
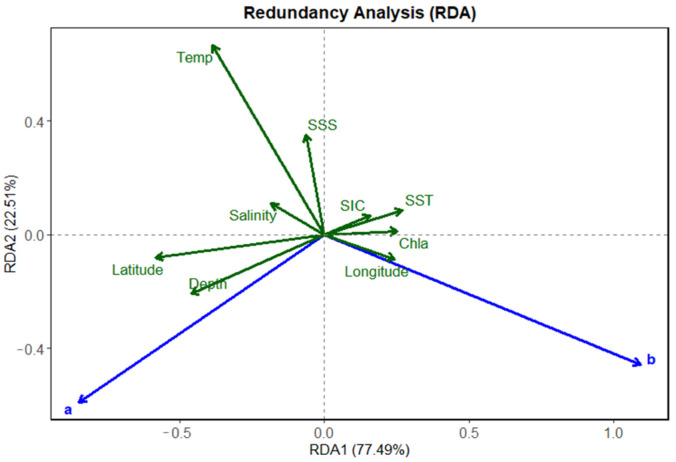
Redundancy analysis (RDA) biplot showing the relationships between environmental variables and the length–weight parameters *a* (condition factor) and *b* (allometric exponent) of ice krill. Blue arrows represent response variables (a and b), and green arrows represent environmental predictors. The first two canonical axes (RDA1 and RDA2) explain 77.49% and 22.51% of the constrained variation, respectively. The plot indicates that a is positively associated with depth, latitude, and salinity, while *b* aligns more strongly with SST, Chl-*a*, SIC, and longitude.

RDA revealed that the first two canonical axes explained 99.99% of the total constrained variation (Figure 7 and Table 2), with RDA1 and RDA2 accounting for 77.49% and 22.51%, respectively. This indicates that the selected environmental and spatial variables possess strong explanatory power for the LWR parameters of ice krill. The condition factor *a* exhibited negative loadings on both RDA1 and RDA2 axes. Along RDA1 axis, *a* was associated with bottom depth, latitude, mean salinity, mean temperature, and SSS, and showed opposition to SST, Chl-*a*, SIC, and longitude. This indicates that *a* is primarily influenced by the vertical hydrographic structure, bathymetry, and latitudinal gradients; In contrast, the allometric exponent *b* showed strong positive loading along the RDA1 axis, closely associated with SST, Chl-*a*, SIC, and longitude. This indicates that *b* is mainly driven by SST, primary productivity, SIC, and regional zonation.

### 3.5. Response of LWR Factors to Environmental and Spatial Gradients by GAM

Based on the RDA results and multicollinearity diagnostics, four key variables were selected for GAM of the LWR parameter *a*: bottom depth, latitude, mean salinity, and mean temperature. These variables showed strong directional associations with *a* in RDA space, and their loadings indicated consistent ecological relevance. Although SSS also exhibited moderate alignment with *a* on RDA1, it was excluded from the final GAM due to partial redundancy with the mean salinity and its relatively weaker explanatory power (Table 2); For the GAM of the LWR parameter *b*, key variables included SST, Chl-*a*, SIC, and longitude. These variables showed strong directional consistency with *b* along the RDA1 axis. All retained variables had VIF values below the standard threshold of 5 (Table 1), confirming low multicollinearity and ensuring model robustness.

**Figure 8:**
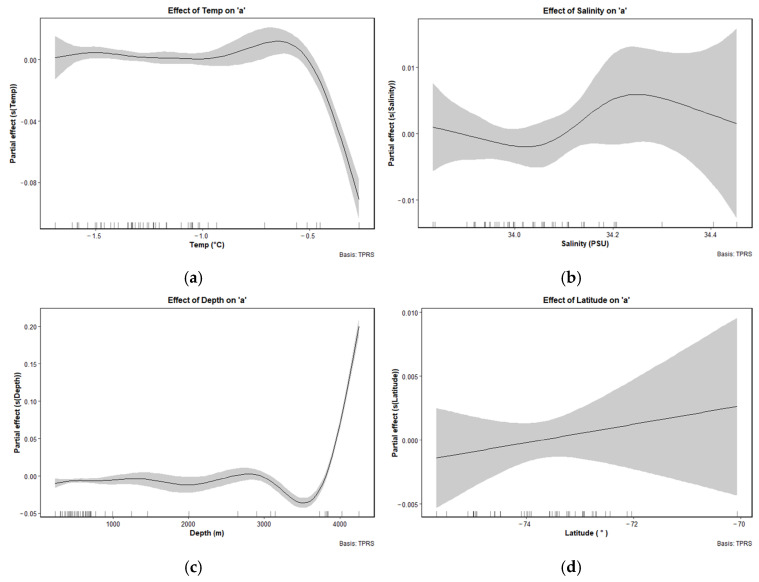
Partial effect plots from generalized additive models (GAM) showing the relationships between the LWR condition factor *a* of ice krill and environmental and spatial predictors: (**a**) mean temperature at 0~200 m; (**b**) mean salinity at 0~200 m; (**c**) bottom depth; (**d**) latitude. Solid black lines represent the fitted smooth functions, and shaded gray areas indicate 95% confidence intervals. Rug marks along the x-axis show the distribution of data points.

The GAM results indicated that bottom depth, latitude, mean salinity, and mean temperature had significant effects on the variation in parameter a (Figure 8). All variables, except latitude, exhibited nonlinear effects. The effect of mean temperature on *a* was relatively stable at lower temperature, but *a* values increased sharply as temperature approached −0.5 °C, followed by a subsequent decline. The effect of mean salinity on *a* followed a unimodal pattern, with peak of *a* values occurring around 34.2 PSU. Bottom depth exhibited a relatively flat or slightly negative effect on *a* at depths shallower than 3500 m, while *a* values increased notably beyond 4000 m. Latitude showed a consistent positive effect on *a* across the range from −74° to −70°.

**Figure 9:**
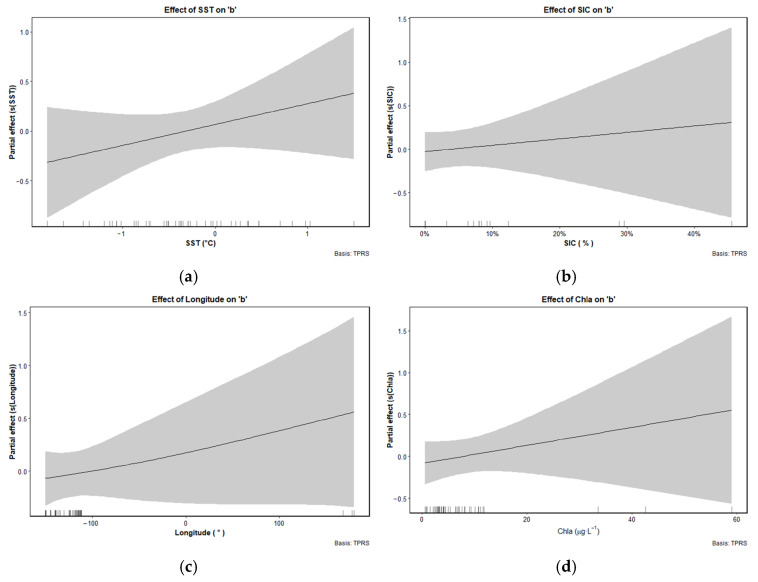
Partial effect plots from generalized additive models (GAM) showing the relationships between the LWR condition factor *b* of ice krill and environmental and spatial predictors: (**a**) sea surface temperature (SST); (**b**) sea ice concentration (SIC); (**c**) longitude; (**d**) chlorophyll-*a* (Chl-*a*). Solid black lines represent the fitted smooth functions, and shaded gray areas indicate 95% confidence intervals. Rug marks along the x-axis show the distribution of data points.

The GAM analysis Indicated that SST, SIC, longitude, and Chl-*a* significantly influenced variation in the LWR parameter *b*, and all exhibiting positive partial effects (Figure 9). Specifically, SST showed a monotonic increase in its effect on *b*, suggesting that higher SST are associated with enhanced structural growth. SIC also showed a positive relationship with *b*, although wider confidence intervals at higher ice concentrations indicated greater model uncertainty in that range. Longitude exhibited a linear positive effect on *b*, with a moderate increase from west to east across the study area. Finally, Chl-*a* showed a clear positive effect on *b*, with increasing effect sizes at higher concentrations, suggesting that areas with greater primary productivity may promote enhanced allometric growth in ice krill.

## 4. Discussion

### 4.1. Regional Variation in the LWR of Ice Krill and Its Ecological Interpretation

The LWR results of ice krill highlight regional heterogeneity in both somatic condition and growth strategies of ice krill populations across the study area. These results across the full dataset revealed a strong allometric growth pattern, with a fitted exponent *b* = 3.137 and a high coefficient of determination. This indicates that, overall, the species exhibits positive allometric growth, where weight increases disproportionately with length, likely reflecting structural development coupled with energy accumulation under favorable environmental conditions. However, when analyzed by region, distinct spatial differences emerged. The estimated *b* values for the Amundsen Sea and the transitional region were 3.151 and 3.187, respectively. Both slightly higher than the full-sample average. In contrast, the Ross Sea exhibited a markedly lower *b* value of 2.858. These patterns suggest that ice krill in Amundsen Sea and transitional region tend to allocate more energy toward structural growth, resulting in relatively greater increases in body length. This trend is consistent with environmental conditions in these regions that may support accelerated development, such as warmer temperatures and higher food availability. Conversely, the sub-isometric growth pattern observed in Ross Sea may reflect an alternative ecological strategy. This could be associated with colder water temperatures, lower primary productivity, or an increased energy investment in lipid reserves rather than somatic elongation. Such regional variation in LWR parameters likely reflect underlying environmental gradients in temperature, productivity, and sea ice cover, which are known to shape krill physiology, metabolic demands, and foraging efficiency. These findings are consistent with previous studies highlighting the importance of habitat characteristics in influencing krill growth dynamics [6,25,26], and underscore the necessity of incorporating spatial context into assessments of population-level growth strategies.

### 4.2. Regional Differentiation in Growth Strategies of Ice Krill

Boxplot analyses revealed distinct regional patterns in the LWR parameters *a* and *b* for ice krill. The condition factor *a* was generally higher in the Amundsen Sea and the transitional zone compared to the Ross Sea, suggesting that ice krill in these areas may allocate more energy toward tissue density or lipid storage. This trend may reflect localized hydrographic conditions, such as proximity to ice-edge phytoplankton blooms or nutrient-enriched upwelling zones, which favor energy accumulation strategies under conditions of food variability or environmental stress [25,27]. The θ-S diagrams suggest that regional bathymetry (shelf vs. slope) and hydrographic structure jointly influence the thermalhaline regime and vertical nutrient flux across these Southern Ocean regions. The transitional zone, situated at the seaward edge of the continental shelf and gradually deepening into the oceanic basin, exhibits unique hydrographic features. In this region, MCDW intrudes at greater depths, and the surface layer is subjected to complex mixing. Elevated *a* values in this area may enhance the capacity of ice krill to store energy in stable, deeper water masses, thereby improving survival prospects during periods of trophic limitation [25,27].

The median values of the allometric growth parameter *b* were similarly high in both the Amundsen Sea and the Ross Sea, indicating strong length-based growth potential in these regions. However, Amundsen Sea exhibited a considerably wider range of *b* values across stations, indicating strong length-based growth potential in these regions. However, the Amundsen Sea exhibited a considerably wider range of *b* values across stations, suggesting greater variability in local somatic growth strategies. This difference may be attributed to the spatial heterogeneity in the upwelling of MCDW. In the Amundsen Sea, MCDW intrudes through deep troughs such as the Pine Island Trough, where localized and variable upwelling leads to heterogeneous nutrient supply conditions across sampling sites [12]. In contrast, the Ross Sea experiences topographically constrained and spatially uniform MCDW intrusion, which may support more consistent g somatic growth patterns among krill populations [28]. Meanwhile, the transitional zone exhibited a noticeably lower median *b* value, suggesting a reduced investment in structural growth and a potential shift toward alternative energy allocation strategies. This divergence likely reflects the distinct environmental heterogeneity across regions. Both Amundsen Sea and Ross Sea are strongly influenced by the MCDW upwelling, which supplies nutrient-rich water and sustained high primary productivity, thereby facilitating somatic growth in euphausiids [12,28]. In contrast, the transitional zone, located at the hydrographic interface between these two systems, is subject to complex mixing of multiple water masses and elevated environmental variability. Such dynamic conditions may disrupt consistent food availability, favoring a conservative growth strategy in krill populations [25]. Moreover, marginal habitat conditions in the transitional zone may promote intra-population variability and metabolic plasticity. The observed lower *b* values reflect a survival-oriented strategy, in which ice krill prioritize maintenance and energy conservation over rapid elongation, enhancing their resilience in response to trophic instability.

A notable discrepancy is evident when comparing the full-sample LWR results with the boxplot analyses at the station level. While the regional LWR fitting suggests a lower *b* value in the Ross Sea, boxplot of station-level *b* values showed that Ross Sea exhibits a relatively high and tightly clustered median *b*, after removing extreme outliers using the IQR method. This difference highlights an important statistical nuance: LWR fitting results are derived from pooled individual data across regions and may be sensitive to the influence of outlier individuals or skewed size distributions [6,29]. In contrast, boxplot analyses summarize central tendencies (e.g., the median) and local variability across sampling stations, yielding a more reliable representation of site-specific growth patterns. Ecologically, this divergence may reflect the combined effects of environmental stability and growth plasticity. The Ross Sea, characterized by colder and more stable hydrographic conditions, may support more uniform krill growth rates across stations, resulting in a narrow but elevated distribution of *b* values [28]. However, the limited presence of large individuals or a narrower range of body lengths may have introduced a downward bias in the estimated LWR slope for this region. Rather than being contradictory, these two analytical perspectives are complementary: the LWR model captures the overarching trend at the individual level, while the boxplot approach provides insight into inter-station consistency. This underscores the importance of integrating multiple analytical scales when evaluating spatial growth variability in euphausiid populations [10]. Nonetheless, it is important to acknowledge that our sampling design across regions was not fully balanced. Specifically, Ross Sea samples were limited to offshore stations near 75°S, while the Amundsen Sea and the transitional zone included both coastal and offshore locations distributed at slightly more northern latitudes. Such spatial disparities may introduce biases due to differing developmental stages, ice krill demographics, and localized oceanographic processes. We therefore advise caution when interpreting cross-basin comparisons, and emphasize that observed differences in LWR parameters could be partially shaped by underlying population composition and regional hydrography. Future work with a more standardized sampling framework across latitudinal and habitat gradients would be valuable to refine these inferences.

In summary, ice krill exhibit regionally divergent growth strategies that are strongly driven by local environmental regimes. Comparable spatial patterns in physiological condition and somatic growth have also been observed in *Euphausia superba* [30] and other polar euphausiids [31], suggesting a broadly conserved adaptive strategy across Antarctic krill species. These consistent responses to environmental gradients underscore the ecological plasticity and resilience of euphausiids inhabiting highly variable Southern Ocean ecosystems.

### 4.3. Environmental and Spatial Drivers of Growth Strategy Divergence in Ice Krill

The RDA results revealed clear patterns in how key environmental and spatial factors influence variation in the LWR parameters of ice krill across the study region. The first two RDA axes explained 99.99% of the total constrained variation, with RDA1 accounting for 77.49% and RDA2 for 22.51%, respectively. These findings demonstrate that the selected environmental and spatial variables possess strong explanatory capacity for the spatial variability in the parameters *a* and *b*, and these results collectively indicate that ice krill exhibits spatially differentiated growth strategies along environmental and geographic gradients in the Southern Ocean. Specifically, *a* tends to be elevated in deeper, colder, and high-latitude environments, reflecting an adaptive strategies focus on energy storage. While *b* is enhanced in warmer, more productive surface waters, indicating greater investment in structural length growth. This variable selection strategy adopted here balances ecological interpretability with statistical robustness, enabling the GAM framework to effectively quantify the nonlinear effects of environmental and spatial gradients on growth parameter of ice krill.

Within the habitat of ice krill, the growth of allometric length (an increase in parameter *b*) typically reflects a physiological response to favorable local environmental conditions. Rising SST are often associated with phytoplankton blooms in spring and summer [32], providing juvenile krill with abundant nutrients that facilitate rapid linear development. Elevated SST also enhances metabolic rates of krill, accelerating digestion, assimilation, and tissue formation [33]. During the austral summer, when water temperatures reach 0–1 °C, both feeding frequency and growth rate increase significantly. This is consistent with the findings of this study, which suggest that surface water temperature promotes enhanced structural growth in Antarctic krill [34,35]. As an indicator of primary productivity and phytoplankton biomass, Chl-*a* concentration is closely linked to the krill nutritional status and somatic growth of krill [2]. Under nutrient-rich conditions, ice krill tend to allocate resources preferentially toward somatic length growth rather than energy storage, which aligns with their ecological strategy of rapid growth and reproduction during late spring and early summer [25,26]. Sea ice also plays a critical role in sustaining phytoplankton productivity. On one hand, melting sea ice releases ice algae and nutrients, triggering surface phytoplankton blooms and extending the nutrient-rich period [36,37]. On the other hand, sea ice cover reduces water column turbulence and enhances stratification within the euphotic zone, thereby supporting higher primary production [38]. Moderate sea ice concentrations provide a physically stable and food-rich feeding environment for ice krill. The observed influence of longitude may reflect regional hydrographic variation. For example, the more easterly regions of the Amundsen Sea is influenced by complex seafloor topography, and experience weaker disturbances from the intrusion of MCDW, helping to maintain high surface productivity [38]. Collectively, this suite of environmental advantages, including elevated temperature, increased food availability, and enhanced ecological stability, supports rapid linear growth in ice krill. This growth pattern leads to higher *b* values as energy is preferentially allocated toward somatic development.

Parameter *a*, by contrast, reflects the physiological condition or energy reserves of ice krill at a given body length. The RDA results indicates that *a* is primarily associated with the temperature-salinity structure of surface water mass, latitude, and bottom depth. The GAM analysis further reveals the complex regulatory influence of environmental and spatial gradients on krill condition. Specifically, mean temperature and mean salinity within the upper 200 m, and bottom depth exhibit nonlinear responses with *a*, whereas latitude shows a significant positive linear effect. Parameter *a* peaks when the mean temperature at 0–200 m approaches −0.5 °C, then decreases sharply, suggesting that krill achieve optimal weight accumulation under cold conditions. At higher temperatures, energy allocation toward somatic reserves may be reduced. This pattern is consistent with an ecological strategy that prioritizes energy storage in cold environments to enhance overwintering survival [39,40]. Similarly, the response of *a* to salinity follow a unimodal curve, with a peak around 34.2 PSU, indicating that moderately high salinity promotes efficient metabolic function and energy storage. However, values of *a* decline beyond this salinity threshold, possibly due to increased osmoregulatory stress under high salinity conditions [41]. An optimal salinity range supports ice krill’s osmoregulation and energy metabolism efficiency, thus influencing parameter *a* [42]. Therefore, when salinity is too high, the osmotic pressure burden on krill increases, leading to energy being directed toward maintaining physiological balance rather than storage, which in turn decreases parameter *a*; The non-linear response of bottom depth on parameter *a* may be related to the regional differences in the invasion depth of MCDW. In shelf regions (such as Amundsen Sea and the Ross Sea), the intrusion intensity of MCDW is relatively weak, but it still provides ample nutrient support for primary productivity. In deeper areas (such as transitional zone), although MCDW intrusion intensity is higher, the surface thermal-salinity stratification is weaker and water column disturbance is stronger, making it difficult for the phytoplankton to maintain stable photosynthetic layers, thus inhibiting effective growth of primary productivity [43,44]. In such nutrient-limited environments, ice krill are more likely to adopt an energy-storage strategy, prioritizing energy for weight accumulation rather than length extension, thereby increasing parameter *a* to enhance environmental adaptability [27,40]; Latitude shows a gradual increase in parameter *a* as the transition occurs from the Antarctic continental shelf (high latitudes) to the outer ocean regions (low latitudes), which may reflect the alignment between the latitude gradient and primary productivity distribution, with low-latitude regions likely providing a more favorable ecological context for condition accumulation in ice krill [2].

## 5. Conclusions

This study provides comprehensive insights into the spatial variability and environmental drivers influencing the length–weight relationship (LWR) parameters of ice krill across the Amundsen Sea and its adjacent regions. Utilizing multi-year survey data (2020–2024) combined with a suite of multivariate statistical analyses, we found that both the condition factor (*a*) and the allometric growth exponent (*b*) exhibited significant spatial differences. These differences were driven by a combination of environmental (e.g., temperature, salinity) and spatial (e.g., latitude, depth) gradients. Boxplot comparisons indicated that krill inhabiting the Amundsen Sea and the transitional waters exhibited higher body condition, whereas individuals in warmer and more productive surface waters showed enhanced structural growth. These findings highlight the region-specific adaptive strategies of ice krill in response to varying hydrographic and ecological conditions and underscore the importance of integrating spatial heterogeneity when evaluating euphausiid growth dynamics in a changing Southern Ocean.

Results from redundancy analysis and generalized additive models revealed that the LWR parameters *a* and *b* exhibit non-linear and region-specific responses to temperature, salinity, and depth, reflecting distinct ecological strategies in different oceanographic contexts. These findings underscore the adaptive plasticity of ice krill growth patterns to heterogeneous environmental conditions and provide a valuable baseline for predicting their responses to ongoing regional climate shifts and ecosystem changes in the Southern Ocean.

## Figures and Tables

**Table 1:** The results of variance inflation factor test (VIF).

Value	SST	SSS	Chl-*a*	SIC	Temp	Salinity	Depth	Latitude	Longitude
*a*	1.684	2.662	1.324	1.505	2.069	3.637	1.906	1.616	2.999
*b*	1.655	2.766	1.311	1.515	2.127	3.531	1.755	1.634	3.108

**Table 2:** Loadings of environmental-spatial variables and biological variables on RDA axes.

Factor	RDA1	RDA2
SST	0.269	0.086
SSS	−0.065	0.351
Chl-*a*	0.251	0.013
SIC	0.159	0.068
Temp	−0.387	0.665
Salinity	−0.184	0.109
Depth	−0.460	−0.207
Latitude	−0.584	−0.080
Longitude	0.242	−0.086
*a*	−0.849	−0.589
*b*	1.093	−0.457

## Data Availability

The biological and environmental data used in this study were obtained during the 36th to 40th Chinese National Antarctic Research Expeditions (CHINARE) and are part of a restricted-access dataset managed by the Polar Research Institute of China (PRIC). Due to national data security regulations and confidentiality agreements, these data are not publicly available. The sea ice concentration data used for supporting environmental characterization were obtained from the National Snow and Ice Data Center (NSIDC) and are freely available at https://nsidc.org/ (accessed on 4 April 2025). No additional publicly archived datasets were generated during the study.
